# Egocentric and Allocentric Spatial Memory in Korsakoff’s Amnesia

**DOI:** 10.3389/fnhum.2020.00121

**Published:** 2020-03-31

**Authors:** Gabriele Janzen, Claudette J. M. van Roij, Joukje M. Oosterman, Roy P. C. Kessels

**Affiliations:** ^1^Behavioral Science Institute, Radboud University Nijmegen, Nijmegen, Netherlands; ^2^Donders Institute for Brain, Cognition and Behavior, Radboud University Nijmegen, Nijmegen, Netherlands; ^3^Centre of Excellence for Neuropsychiatry, Vincent van Gogh Institute for Psychiatry, Venray, Netherlands; ^4^Centre of Excellence for Korsakoff and Alcohol-Related Cognitive Disorders, Vincent van Gogh Institute for Psychiatry, Venray, Netherlands; ^5^Department of Medical Psychology, Radboud University Medical Center, Nijmegen, Netherlands

**Keywords:** Korsakoff’s syndrome, spatial memory, landmarks, delay, egocentric, allocentric, amnesia

## Abstract

The goal of the present study was to investigate spatial memory in a group of patients with amnesia due to Korsakoff’s syndrome (KS). We used a virtual spatial memory task that allowed us to separate the use of egocentric and allocentric spatial reference frames to determine object locations. Research investigating the ability of patients with Korsakoff’s amnesia to use different reference frames is scarce and it remains unclear whether these patients are impaired in using ego- and allocentric reference frames to the same extent. Twenty Korsakoff patients and 24 matched controls watched an animation of a bird flying in one of three trees standing in a virtual environment. After the bird disappeared, the camera turned around, by which the trees were briefly out of sight and then turned back to the center of the environment. Participants were asked in which tree the bird was hiding. In half of the trials, a landmark was shown. Half of the trials required an immediate response whereas in the other half a delay of 10 s was present. Patients performed significantly worse than controls. For all participants trials with a landmark were easier than without a landmark and trials without a delay were easier than with a delay. While controls were above chance on all trials patients were at chance in allocentric trials without a landmark present and with a memory delay. Patients showed no difference in the ego- and the allocentric condition. Together the findings suggest that despite the amnesia, spatial memory and especially the use of ego- and allocentric reference frames in Korsakoff patients are spared.

## Introduction

Spatial memory is extremely important for successful navigation through our environment. Therefore, information about landmarks, spatial locations and routes have to be processed efficiently. Locations of landmarks can be determined in two fundamental ways to allow successful navigation and orientation; by egocentric and allocentric reference frames (e.g., O’Keefe and Nadel, 1978; Klatzky, 1998; van den Brink and Janzen, 2013). Egocentric coding involves the representation of positions of objects in relation to the observer’s body (subject-to-object). This system can be used when the observer is not moving or when he/she can track his/her movements based on optic flow, vestibular and proprioceptive cues. The second system, allocentric coding, involves an externally referenced spatial coding based on inter-object relations to determine the location of an object (object-to-object). Allocentric coding is independent of the observer’s current position. In adults, there is growing evidence for a parallel spatial-representational system of these two different coding types (Simons and Wang, 1998; Wang and Simons, 1999; Committeri et al., 2004; Mou et al., 2004; Nadel and Hardt, 2004; Burgess, 2006; Waller and Hodgson, 2006, see Ekstrom et al., 2014 for a critical review on the neural correlates of allocentric spatial representations).

Several disorders and syndromes are known to have impaired episodic memory functions, including spatial memory dysfunction, as one of the symptoms. Despite their memory deficits, patients with amnesia can also have spared spatial memory aspects (Kessels et al., 2011; Oudman et al., 2011; see Rosenbaum et al., 2015 for a case study with a developmental amnesia patient showing preserved as well as impaired spatial memory). Previous research findings have shown for example impaired allocentric spatial memory functions in patients with Alzheimer’s dementia or mild cognitive impairment, while egocentric spatial memory seems to be spared (Hort et al., 2007; Iachini et al., 2009). Difficulties in allocentric processing have also been observed in normal aging (Moffat and Resnick, 2002; Iaria et al., 2009; Wiener et al., 2012, 2013). Harris et al. (2012) observed a more specific decline showing that aging impairs switching from an egocentric to an allocentric strategy while switching to an egocentric strategy remained unaffected. Together these findings show that especially the allocentric reference frame is challenging not only for patients with memory deficits but also for healthy elderly (see Lester et al., 2017 for a review on spatial cognition in normal and impaired aging).

Another group of patients who are known to suffer from memory deficits, but also have spared memory capacities, are patients with Korsakoff’s syndrome (KS); patients with this syndrome all display excessive memory disorders, lack of binding abilities, attention deficits and disorientation in time and place due to excessive alcohol abuse in combination with vitamin B1 deficiency (Kopelman, 2002; Tielemans et al., 2012; Arts et al., 2017; see Heirene et al., 2018 for a systematic review on the assessment of alcohol-related cognitive impairment including KS). Spatial memory performance in patients with this syndrome is characterized by a deficit in explicitly remembering spatial information (Holdstock et al., 1999; Kessels et al., 2000; van Asselen et al., 2005; Postma et al., 2006, 2018; Kessels and Kopelman, 2012).

To date, reports exploring the ability of amnesic patients with KS to use the different object-location-framing types are scarce, but a previous study by Holdstock et al. (1999) focused on this process. They designed a spatial memory task in which participants should recall the position of single spot LED lights after various delays. Three task conditions were used, a short-delay condition (0, 3 or 8 s), as well as an allocentric and egocentric condition (both with delays of 5, 20 or 60 s). In the short-delay condition, the participant was instructed to look away from the board, without changing position. In the allocentric condition, the participant had to move around the light-board during the delay. Due to the enlightened room during this procedure, participants could make use of external stimuli to re-orientate. The egocentric condition took place in a dark room where participants could not make use of external stimuli and could only rely on his/her body position. Participants were encouraged not to move during this condition. The results showed that patients with amnesia due to KS were impaired in both the ego- and allocentric condition to the same extent. In both conditions, patients’ performance declined to a greater extent due to the extension of the delay as compared to the performance of the controls. Due to this accelerated forgetting, it was concluded that the KS patients have impaired memory for both allocentric and egocentric information.

The present study aimed to investigate the ability of patients with KS to use egocentric and allocentric frames of reference to determine object locations. While previous studies have shown an impairment particularly in allocentric processing with spared egocentric memory function in healthy aging (Moffat and Resnick, 2002; Iaria et al., 2009; Wiener et al., 2012, 2013) as well as in patients with memory deficits (Hort et al., 2007; Iachini et al., 2009), Holdstock et al. (1999) observed impairment in both allocentric as well as egocentric processing in KS patients. The present study aimed to shed light on these diverse findings.

Here, we extended the previous work by Holdstock et al. (1999) by using an ecologically valid paradigm. Furthermore, we examined the effect of adding landmarks, as this may facilitate allocentric representations and contributes to re-orientation (Learmonth et al., 2002). To further clarify the ability of these patients to use egocentric and allocentric strategies, the current study applied a paradigm previously designed by van den Brink and Janzen (2013). In their study, a group of 30 to 35-month-old toddlers watched an animation of a bird flying in one of two trees standing in a virtual environment. After the bird disappeared, the camera turned around, by which the trees were briefly out of sight and then turned again to the center of the environment in which the trees were located. Participants were asked in which tree the bird was hiding. In half of the trials, a landmark was shown. Comparable to Holdstock et al. (1999), and in addition to the paradigm of van den Brink and Janzen (2013), the present study made use of a direct condition and of a delay condition in which the camera turn is delayed by 10 s.

In addition to the studies of Holdstock et al. (1999) and van den Brink and Janzen (2013), the current study explored if the performance on this virtual spatial memory task (the “bird task” of van den Brink and Janzen, 2013) was related to the performance on an everyday memory test (the Global Memory Index of the Rivermead Behavioral Memory Index—Third Edition; RBMT-3). Since mental rotation is of great importance in the present paradigm, we also studied whether the participants were able to mentally rotate spatial information and to which degree the performance on this paper-and-pencil mental rotation task was related to the performance on the bird task.

Concerning the underlying neural correlates, the hippocampus is crucial in using allocentric frames of reference (e.g., O’Keefe and Nadel, 1978; Maguire et al., 1998; Holdstock et al., 2000). Although KS is primarily characterized by diencephalic lesions (Aggleton and Saunders, 1997; Arts et al., 2017), hippocampal atrophy has been reported in these patients as well (e.g., Sullivan and Pfefferbaum, 2009). Furthermore, damage to diencephalic structures that are connected to the hippocampus may also result in impaired allocentric representations (Holdstock et al., 1999). Holdstock et al. (1999) not only observed an impairment in the allocentric condition, but also in egocentric processing. Similarly, we expect that KS patients perform worse in all conditions as compared to healthy controls. We furthermore hypothesize that KS patients have a preference for an egocentric strategy and will perform worse in conditions where only an allocentric strategy will be successful (Neave et al., 1997; Kopelman, 2002). Since, an egocentric representation alone is not providing enough information to correctly perform the task, we hypothesize that the amnesic patients will make more egocentric errors (i.e., selecting the position of the tree the bird was hiding in before the turn) in comparison to the controls. Although landmarks facilitate the use of an allocentric object-location strategy, we expect the patients not to benefit from these to the same extent as controls. Concerning the findings of Holdstock et al. (1999) and the memory deficits KS patients have (Kopelman, 2002; Kessels and Kopelman, 2012), we expect the performance of the patients to decline more than the control performance after a delay.

## Materials and Methods

### Participants

Twenty patients with severe anterograde amnesia, diagnosed with KS and 24 healthy age- and intelligence matched controls (see Table 1) successfully participated in the present study and were included in the analyses. Four more patients did not want to complete the task and two patients did meet the exclusion criteria; their data was not included in the further analyses. General exclusion criteria for both groups were a stroke in history, other neurological disorders, like alcohol-related dementia, premorbid intelligence level below 65 and not being able to communicate in Dutch. All patients were abstinent for at least 6 weeks before being tested. All patients were recruited from the Centre of Excellence for Korsakoff and Alcohol-Related Cognitive Disorders of Vincent van Gogh Institute for Psychiatry, Venray, The Netherlands. Inclusion criteria for the patients were a DSM-5 diagnosis of a Major Neurocognitive Disorder due to Alcohol, Amnestic/Confabulatory Type (confirmed by neuropsychological assessment, neurological examination, and neuroradiological findings) and meeting the criteria for the KS (Kopelman, 2002; Arts et al., 2017). Available MRI scans were visually rated to exclude other diseases by an experienced researcher, focusing on global cortical (GCA) and medial temporal lobe atrophy (MTA; see Wahlund et al., 2000) as well as white-matter hyperintensities (WMH; Fazekas et al., 1993). MRI data were available for fifteen patients; five patients did not undergo an MRI-scan. The matched healthy volunteers were recruited from the staff of the clinic or through relatives of one of the researchers.

**Table 1:** Demographical and other characteristics for both groups.

Characteristic	Healthy controls	Korsakoff’s amnesia	Statistic	*p*-value
Number of participants	24	20	-	-
Sex (men:women)	19:5	16:4	$\chi_{\left(1\right)}^{2}$ = 0.005	*p* = 0.946
Age (*M, SD*)	57.79 (6.90)	60.20 (7.70)	*t*_(42)_ = 1.095	*p* = 0.280
Educational level (*Mode, range)*	5 (2–7)	3 (2–7)	*Mann–Whitney U* = 164.500	*p* = 0.066
Intelligence level estimation (NART IQ; *M, SD*)	89.96 (11.51)	89.65 (15.84)	*t*_(42)_ = −0.075	*p* = 0.941
Mental Rotation Task (*M, SD*)	9.42 (2.62)	7.10 (1.68)	*t*_(42)_ = −3.46	*p = 0*.001
RBMT-3 Global Memory Index (*M, SD*)	-	59.79 (5.86)	-	-
MTA		0: *N* = 8		
		1: *N* = 3		
		2: *N* = 3		
		3: *N* = 1		
		4: *N* = 0		
		NA: *N* = 5		
GCA		0: *N* = 2		
		1: *N* = 9		
		2: *N* = 3		
		3: *N* = 1		
		NA: *N* = 5		
WMH		0: *N* = 2		
		1: *N* = 11		
		2: *N* = 2		
		3: *N* = 0		
		NA: *N* = 5		

*Notes. NART, National Adult Reading Test; RBMT-3, Rivermead Behavioral Memory Test—Third Edition; MTA, Medial Temporal Lobe Atrophy Rating: 0 = no atrophy, 1 = widening of choroid fissure, 2 = widening of choroid fissure and temporal horn of lateral ventricle, 3 = moderate hippocampal volume loss, 4 = severe hippocampal volume loss; GCA = Global Cortical Atrophy Rating: 0 = no atrophy, 1 = opening of sulci and mild ventricular enlargement, 2 = volume loss of gyri and moderate ventricular enlargement, 3 = knife-blade atrophy and severe ventricular enlargement; WMH = Fazekas White-Matter Hyperintensities Rating: 0 = no WMH, 1 = pencil-thin periventricular or punctuate focal deep WMH, 2 = smooth halo periventricular or early confluence of focal deep WMH, 3 = irregular periventricular WMH extending into the deep white matter or large confluent regions of deep WMH*.

In all participants, premorbid intelligence was estimated using the Dutch version of the National Adult Reading Task (NART; Schmand et al., 1992). Additionally, the Mental Rotation Task (MRT; Shepard and Metzler, 1971; Aleman et al., 2004) was administered. In this paper-and-pencil task, consisting of seven items, the participant is asked which two of four turned block-patterns is the same as the target pattern. The other two patterns are a mirrored version of the target. During the explanation of this task, we made use of two 3D demonstration pieces to clarify the rotation of the patterns.

Besides, the KS patients completed the Rivermead Behavioral Memory Task—Third edition (RBMT-3; Wilson et al., 2008), an ecologically valid episodic memory test battery. Education level was classified on a 7-point-scale based on the Dutch educational system (Verhage, 1964). Table 1 shows the demographical and behavioral characteristics of both groups. Note that the healthy controls had a slightly higher education level compared to the patients, but no group differences were found for estimated premorbid intelligence (see Table 1).

### Spatial Memory Task

A computerized paradigm, developed by van den Brink and Janzen (2013), was adopted and used to study spatial memory. Commercially available animation suited software Blender^1^ was used to construct 48 movies, which were shown by the software Presentation. Each movie lasted 30 s in the direct condition and 40 s in the 10-s delay condition. The movies showed an animated bird, appearing in front of the camera, turning around and flying into one of three identical trees. Other than in the original experiment designed by van den Brink and Janzen (2013) in which two trees were shown, we here used environments in which three trees were positioned to increase the difficulty of the task. Each tree was positioned at different distances within an open 3D environment, forming an equilateral triangle. After the disappearance of the bird, the camera perspective followed a path that resulted in a perspective change; 90° to the left or 90° to the right of the center of the environment. This change in perspective led to the illusion of self-motion by the participant. During this turn, the trees and all other objects were out of sight for a while, preventing tracking of the bird’s hiding place. While the camera turned away the empty landscape without trees and landmark was shown. In the delay condition, the empty landscape was shown for 10 s and in the direct condition, the camera turned back to the center of the environment directly after turning away. At the end of the turn path, the camera again turned to the center of the environment which led to the reappearance of all objects. The total duration of the turn was 4 s. In 24 movies, the turn that led to the perspective change, was delayed by 10 s. The distance to the center of the environment (before and after the spatial transformation) was six Blender units (6 m). The distance of the spatial transformation was 8.5 m. After the reappearance of all objects in all conditions, the participants should point to the tree in which they believe the bird was hiding.

**Figure 1:**
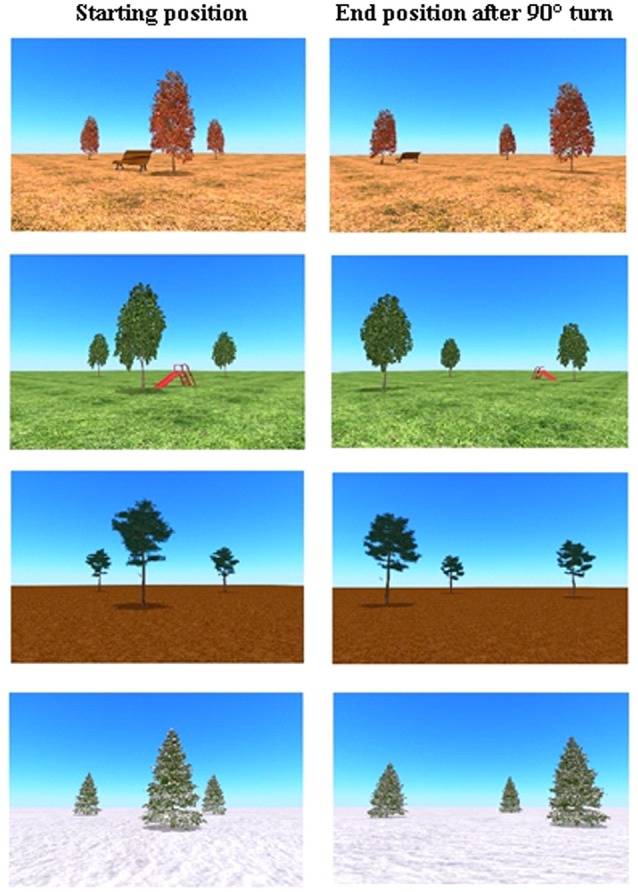
Overview of the four environments, with and without landmark and a reproduction of the spatial transformation in the virtual spatial memory task. The direction of the turn is respectively right, left, left, right.

**Figure 2:**
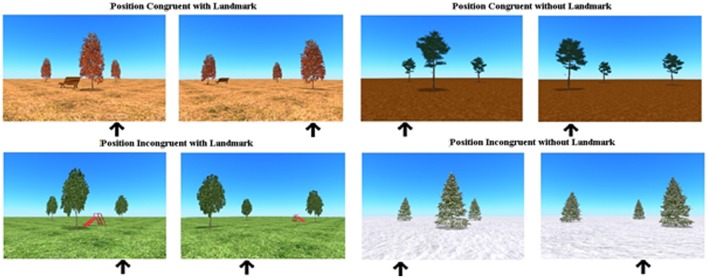
Overview of position congruent (ego- as well as allocentric strategies are successful) and position incongruent trials (only allocentric strategy is successful), with and without a landmark in the virtual spatial memory task.

The trees were positioned in four 3D environments: snow, autumn, mud and a grass landscape. In the environments of autumn and grass, a landmark was added; respectively a bench and a slide. The landmark was positioned inside the cluster of the three trees, but closer to the front tree (see Figure 1). The presence of a landmark possibly facilitated the use of an allocentric strategy (object-to-object relation use). To survey subject-to-object relation use, in one-third of the trials the starting position of the correct tree, in which the bird was hidden, corresponded to the position of that tree relative to the participant’s body after the camera angle change (for example before and after the turn, the tree in which the bird was hidden, was at the most right side relative to the participant). These trials that allow the use of an egocentric strategy as well as the use of an allocentric strategy were called position-congruent trials. In all the other trials, the position of the correct tree before the turn did not match the position of that tree, relative to the participant’s body, after the turn (position-incongruent trials). These trials only allow the use of an allocentric strategy to be successful (see Figure 2). In this position-incongruent trials, an egocentric choice resulted in an incorrect response; only an allocentric representation led to the correct response in these trials.

Consequently, all factors led to the distribution of the movies/trials into eight conditions: (1) position congruent, delay, landmark; (2) position congruent, delay, no landmark; (3) position congruent, direct, landmark; (4) position congruent, direct, no landmark; (5) position incongruent, delay, landmark; (6) position incongruent, delay, no landmark; (7) position incongruent, direct, landmark; and (8) position incongruent, direct, no landmark. The position congruent conditions each consisted of four movies; position incongruent conditions each consisted of eight movies. Alternately, a block of 12 trials with a delay and 12 trials with a direct recall was shown. Whether the participant began with a block with or without delay was counterbalanced.

### Procedure

The total duration of the procedure was 45–60 min and it took place in a quiet room in the Korsakoff Clinic in Venray or the participant’s environment. The procedure started with the paper-and-pencil MRT, which took 10 min. After this, the participants were seated in front of a 16.7-inch laptop, which was within arm reach of the participant, for the start of the bird-task. Participants were told that they were going to see an animation of a bird flying away and hiding in one of three trees and that they should watch carefully in which tree the bird flew and to remember this place during a spatial transformation of the camera. They were also told that in half of the movies, this turn of the camera was delayed so that they should remember the position of the bird for a longer time. After the spatial transformation, the participant was required to indicate where the bird was hiding; they could respond verbally or they could point to the tree in which they thought the bird was hiding. After the administration of this response by the researcher, the bird reappeared from the correct tree, giving the participant feedback. Participants were given two practice trials in advance: one with a direct recall and one with a delay. The study was approved by the Institutional Review Board of Vincent van Gogh Institute for Psychiatry (CWOP27/1/2014) and written informed consents were obtained, as per the Declaration of Helsinki.

### Analyses

The total percentage of correct responses for each condition was measured. As each trial has three response options, the chance level performance was indicated at 33.3% correct answers. An alpha of 0.05 was used in all analyses. Effects with an alpha between 0.05 and 0.10 were judged marginally significant. First, we compared both groups using a 2 (Group: KS vs. controls) × 2 (Position: incongruent vs. congruent) × 2 (Delay: direct vs. delay) × 2 (Landmark: yes vs. no) repeated measures ANOVA. Participants could respond in an allocentric way (which leads to the correct answer), in an egocentric way (which leads to incorrect responses in position incongruent trials) or in a random way. *T*-tests were used to compare the amount of egocentric errors in both groups.

Furthermore, it was calculated if both groups differ in their performance on the MRT and if they performed above chance level of seven correct answers (max = 14). Also, we correlated the spatial memory performance with the performance on the RBMT-3 and the MRT and the visually rated MTA, and GCA.

## Results

**Figure 3:**
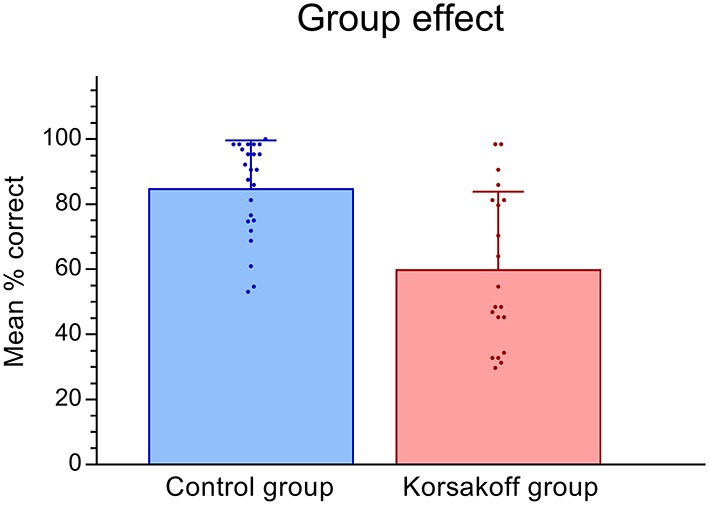
Means and standard deviations for Korsakoff’s patients vs. controls. Scatter dots show individual mean performance for all conditions.

**Figure 4:**
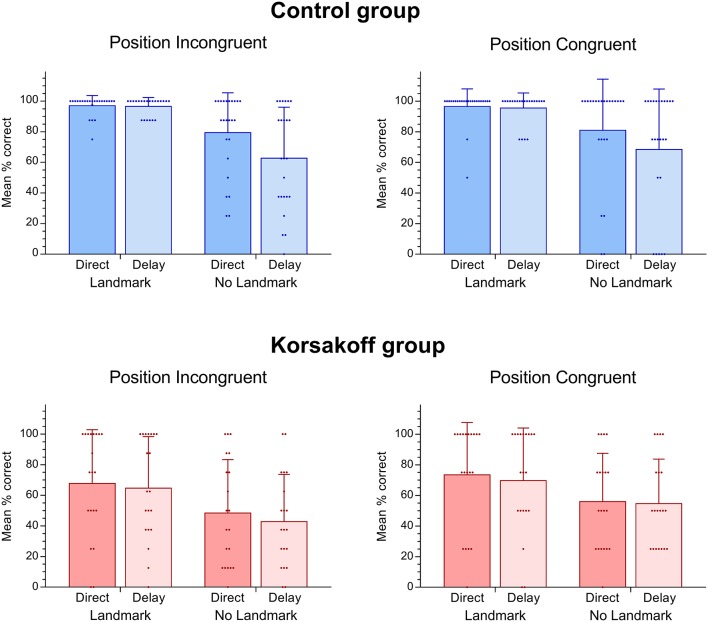
Means and standard deviations as well as scatter dots of individual performance in the virtual spatial memory task for both controls and patients with Korsakoff’s syndrome (KS).

Figures 3, 4 show the performance on the spatial memory task. Controls performed above chance level on all eight conditions (all *p*-values < 0.05). Patients performed at chance in the position incongruent condition without a landmark and with a delay (*p* = 0.16). Performance on all other conditions were above chance (*p* < 0.05, Figure 4). This analysis revealed that, in general, patients performed significantly worse (*M* = 60.0, *SE* = 4.33) than controls (*M* = 84.95, *SE* = 3.95; *F*_(1,42)_ = 18.15, *p* < 0.005, $\eta_{\text{p}}^{2}$ = 0.30, Figure 3). In addition, a significant performance difference between trials with and without a landmark was found (*F*_(1,42)_ = 27.24, *p* < 0.005, $\eta_{\text{p}}^{2}$ = 0.39), with participants scoring higher on the trials with a landmark (*M* = 82.97, *SE* = 3.05) than on the trials without landmark (*M* = 61.98, *SE* = 3.99). Furthermore, a significant main effect of Delay was found (*F*_(1,42)_ = 6.36, *p* = 0.02, $\eta_{\text{p}}^{2}$ = 0.13), with participants scoring higher on trials with a direct recall (*M* = 75.25, *SE* = 3.01), than on trials with a delay (*M* = 69.70, *SE* = 3.24). The main effect of Position type was not significant (*F*_(1,42)_ = 3.36, *p* = 0.07, $\eta_{\text{p}}^{2}$ = 0.07). There the performance on position-congruent trials (*M* = 74.71, *SE* = 3.12) was only slightly higher compared to position-incongruent trials (*M* = 70.23, *SE* = 3.20). The Landmark × Delay interaction effect was not significant (*F*_(1,42)_ = 2.92, *p* = 0.095, $\eta_{\text{p}}^{2}$ = 0.07). Furthermore, the 3-way interaction of Landmark × Delay × Group was not significant (*F*_(1,42)_ = 2.92, *p* = 0.095, $\eta_{\text{p}}^{2}$ = 0.07).

Neither the interaction between Group and Landmark nor the interaction between Group and Delay were significant (*F*_(1,42)_ = 0.40, *p* = 0.53 and *F*_(1,42)_ = 0.92, *p* = 0.34, respectively), nor was the Group by Position interaction (*F*_(1,42)_ = 1.53, *p* = 0.22), the Position by Landmark interaction (*F*_(1,42)_ = 1.19, *p* = 0.28) or the Position by Delay interaction (*F*_(1,42)_ = 0.26, *p* = 0.61). Furthermore, none of the other 3- or 4-way interactions were statistically significant (all *p*-values > 0.27).

Additional analyses for the separate groups were performed, showing a strong interaction effect of Landmark and Delay in the control group (*F*_(1,23)_ = 8.35, *p* = 0.008, $\eta_{\text{p}}^{2}$ = 0.27), which was absent in the KS group (*F*_(1,19)_ = 0.00, *p* = 1.00), indicating that in controls a delay resulted in a worse performance on the no-landmark trials as compared to the landmark trials. In both groups, a significant main effect for Landmark was found (Patients: *F*_(1,19)_ = 9.78, *p* = 0.006, $\eta_{\text{p}}^{2}$ = 0.34; Controls: *F*_(1,23)_ = 18.63, *p* = 0.001, $\eta_{\text{p}}^{2}$ = 0.45). A significant main effect of Delay was observed in the controls only (*F*_(1,23)_ = 7.84, *p* = 0.01, $\eta_{\text{p}}^{2}$ = 0.25; KS: *F*_(1,19)_ = 0.95, *p* = 0.34). In neither of the two groups did we find a significant main effect for Position type or any significant 2- or 3-way interactions with Position type (all *p* > 0.10).

A *t*-test to compare the percentage egocentric errors in both groups did not reveal a group difference (*p* = 0.16). Correlational analyses showed that better performance on the bird task was strongly correlated with better performance on the MRT (*r* = 0.41, *p* = 0.005). Separate correlations for both participant groups were not significant. A Spearman rank correlation on the MRI measures revealed a not significant correlation between the amount of MTA and the performance on the bird task (*r* = −0.492, *p* = 0.062) as well as between the amount of GCA and performance on the bird task (*r* = −0.216, *p* = 0.440). The correlation between RBMT-3 performance and spatial memory performance was not significant (*p* > 0.19).

## Discussion

The present study aimed to investigate the ability of KS patients to use egocentric and allocentric frames of reference to remember an object’s location. In a virtual reality environment, amnesic patients and healthy controls should determine the hiding place of an animated bird with and without a delay of 10 s as well as with and without a landmark present. Position incongruent trials needed to be processed in an allocentric frame of reference while position congruent trials could be solved with the use of an egocentric frame of reference. Results showed that patients generally performed significantly worse than controls. While controls performed above chance level on all trials, patients were at chance in position-incongruent trials without a landmark present and with a memory delay. As expected for all participants trials with a landmark were easier than without a landmark and trials without a delay were easier than with a delay. A trend was observed with position congruent trials being easier than position incongruent trials. An interaction effect with the factor group involved showed that controls benefited more from a landmark and the no-delay condition than patients.

Results did not show any differences between cognitively unimpaired controls and Korsakoff amnesics in their performance on position incongruent trials in comparison to position congruent trials. Furthermore, the performance of the patients on position-congruent and position-incongruent trails did not differ. This shows that the performance of patients with Korsakoff’s amnesia does not decline more in a task in which an allocentric frame of reference is needed relative to a task in which an egocentric strategy is sufficient. Besides, adding a landmark improved the performance of the patients, which indicates their use of an allocentric strategy. Furthermore, the amount of egocentric errors did not differ between healthy controls and Korsakoff amnesics. Together these results indicate, in comparison to our hypotheses, the efficient use of an allocentric frame of reference to determine object locations by patients with Korsakoff amnesia. This finding is in line without results from Holdstock et al. (1999) that showed that patients with KS had a comparable impairment on both ego- and allocentric conditions.

In general, all participants’ achievements declined due to the addition of a delay. However, the controls only showed a delay effect in the condition without a landmark; for an optimal delay performance, a landmark was helpful for them. The patient group alone did not show a delay effect. This might be because trials without an extra delay of 10 s involved a memory component. After all, the hiding place of the bird needed to be remembered for the short period in which all objects were out of sight. Therefore, possibly the difference between both conditions was too small to detect a memory decline in the patient group.

All participants benefited from a landmark being present. Landmarks are helpful in navigation because they may facilitate the use of an allocentric reference frame and contribute to re-orientation in an environment (Learmonth et al., 2002; Janzen and Jansen, 2010; Eppstein and Vass, 2013). However, findings in toddlers with the “bird task” developed by van den Brink and Janzen (2013) showed that young children did not benefit from the presence of landmarks. On the contrary, children were distracted and even performed worse in trials with landmarks that were of interest to children. Young children seem to rely on optic flow cues only, whereas adults prefer a landmark strategy which might be beneficial in trials that require an allocentric strategy. Other than the patients and controls in the present study who did not show a difference between position-congruent and incongruent trials, 30-month old children in the study by van den Brink and Janzen (2013) demonstrated a strong decline in position incongruent trials. Most likely successful landmark use also requires the ability to use an allocentric strategy.

Further analyses show that better performance on the bird task strongly correlates with better performance on the paper-and-pencil mental rotation task (Shepard and Metzler, 1971). Both participants groups showed similar relationships which were not significant for the separate groups, most likely due to the low number of participants. This finding shows that the ability to mentally rotate objects also links to reorientation skills in larger environments. Furthermore, although patients with more hippocampal atrophy performed worse on the bird task, the results on the RBMT-3 and bird task were not correlated. This—in combination with the correlation between the bird task and mental rotation—supports the notion that the effects we have found with our paradigm do not only reflect the effect of the overall amnesia but may be the result of a specific deficit in the processing of spatial orientation related information.

It is worthwhile to notice that the patient group with 20 Korsakoff patients could be seen as relatively small. However, studies with larger groups are sparse as described in the review on implicit memory by Hayes et al. (2012). The number of patients in the present study, in addition, does not differ from previous studies examining spatial memory (e.g., Oudman et al., 2016). Nevertheless, a replication in a larger sample would be valuable. A further limitation is that the present study includes a visual rating of the MRI scans for a subset of 15 patients only. Future research in a larger sample including a more precise method to measure hippocampal volume, such as voxel-based morphometry, would allow for concrete conclusions about the relationship between behavioral performance and brain regions. A future study could additionally consider to include more trials per condition. A larger number of trials might reduce the variability in the data. However, a new study design should balance between more trials and possible fatigue and motivation problems in the patient group, since Korsakoff’s amnestic have severe cognitive impairments.

Since KS is more common in men than in women our participant sample has a corresponding unequal distribution which makes analyses including sex not informative and beyond the scope of our manuscript. However, sex-related differences in spatial navigation tasks are a matter of debate, with often men outperforming women in navigation tasks (see e.g., Coutrot et al., 2018), whereas women often have an advantage in object location tasks (Murphy et al., 2009; Bocchi et al., 2020; but see also Postma et al., 2004). Note, however, that the effect sizes for sex difference on cognitive tasks are typically small, and that the effects of the amnesia itself overshadow these subtle sex differences.

In sum, our study confirms a spatial memory deficit in KS patients with the patient group performing at chance in the most difficult condition (position incongruent trials, without landmark and with memory delay). In contrast to previous findings in young children both participant groups benefited from a landmark present. In line with findings by Holdstock et al. (1999), KS patients showed no difference in the ego- and the allocentric condition, suggesting that patients can efficiently use an allocentric frame of reference to maintain orientation in a spatial environment. Our study suggests that despite the amnesia and in line with findings by Oudman et al. (2016), spatial memory in KS patients can be spared.

## Data Availability Statement

The datasets generated for this study are available on request to the corresponding author.

## Ethics Statement

The study was approved by the Institutional Review Board of the Vincent van Gogh Institute for Psychiatry (CWOP #27-01-2014) and written informed consents were obtained in accordance with the Declaration of Helsinki.

## Author Contributions

GJ and RK planned and designed the study, with input from CR. CR collected and coded the data with input from JO. GJ, RK, and CR analyzed the data. GJ, CR, and RK drafted and wrote the manuscript. JO provided input on the drafted version of the manuscript.

## Conflict of Interest

The authors declare that the research was conducted in the absence of any commercial or financial relationships that could be construed as a potential conflict of interest.

## Footnotes

^1^www.blender.org
